# Long-Term Changes in Intraocular Pressure after Vitrectomy for Rhegmatogenous Retinal Detachment, Epi-Retinal Membrane, or Macular Hole

**DOI:** 10.1371/journal.pone.0167303

**Published:** 2016-11-29

**Authors:** Kentaro Yamamoto, Takeshi Iwase, Hiroko Terasaki

**Affiliations:** Department of Ophthalmology, Nagoya University Hospital, Nagoya, Aichi, Japan; Massachusetts Eye & Ear Infirmary, Harvard Medical School, UNITED STATES

## Abstract

**Purpose:**

To determine the long-term changes in the intraocular pressure (IOP) following vitrectomy for rhegmatogenous retinal detachment (RRD), epiretinal membrane (ERM), and macular hole (MH), and to investigate the relationship between the retinal disease and the incidence of late-onset IOP elevation.

**Methods:**

This was a retrospective, observational, comparative study. We reviewed the medical records of 54 eyes of 54 RRD patients, 117 eyes of 117 ERM patients, and 75 eyes of 75 MH patients who underwent 20-, 23- or 25-gauge vitrectomy. The IOPs before surgery and 1, 3, 6, and 12 months following vitrectomy, and also at the final visit (average, 23.95 months) were evaluated. We defined a significant increase in the IOP as an increase of ≥4 mmHg from the preoperative IOP, and this increase was taken to be a ‘death’ event for the Kaplan-Meier survival analyses.

**Results:**

The mean follow-up period was not significantly different among the groups. The mean IOP at 3 (*P* = 0.001) and 12 (*P* = 0.011) months following the vitrectomy and at the final visit (*P* = 0.002) were significantly higher than that before the vitrectomy in the RRD group. The mean IOP in the RRD group was significant higher than that in the ERM group at 1 (*P* = 0.005), 3 (*P* = 0.009), and 12 (*P* = 0.013) months following vitrectomy, and at the final visit (*P* = 0.032). Kaplan-Meier survival analyses showed that the RRD group had a significantly higher risk of an IOP increase following vitrectomy than the other groups (*P*<0.001 by log-rank test). Multivariate logistic regression analyses showed that a preoperative diagnosis of RRD was the only risk factor that was significantly associated with a postoperative IOP elevation after excluding eyes with a low preoperative IOP (odds ratio, 3.208; *P* = 0.003).

**Conclusions:**

A late-onset IOP elevation following vitrectomy was observed only in eyes that underwent RRD surgery. The elevation was probably caused by the specific characteristics and surgical procedures of RRD. Clinicians should pay more attention to the IOP elevation for long times after vitrectomy especially in eyes with RRD.

## Introduction

Pars plana vitrectomy is performed to treat various kinds of vitreoretinal diseases.[1–4] An early postoperative transient increase in the IOP is not an uncommon complication because of inflammation, hemorrhage, use of viscoelastic materials, silicone oil tamponade, and long-lasting gas tamponade.[5–8] However, whether there is an elevation of the IOP in the long term after vitrectomy is still controversial.

Some studies have shown a late-onset IOP elevation. [9] [10] [11] Chang first reported the development and worsening of open angle glaucoma (OAG) during a long-term period following vitrectomy compared to the fellow eyes.[9] He hypothesized that an increase in the partial pressure of oxygen in the vitreous cavity after vitrectomy can cause oxidative stress on the trabecular meshwork resulting in an IOP elevation which is exacerbated by lens extraction. Koreen et al. reported that lens extraction was a strong risk factor for the development of late-onset OAG after uncomplicated PPV [10], and Toyokawa et al. also reported an IOP elevation after vitrectomy in pseudophakic eyes. [11]

In contrast, Lalezary et al. and Mi et al. reported that vitrectomy did not cause an IOP elevation in the long-term in eyes with ERM or MH.[12, 13] Yu et al. reported that OAG did not develop after vitrectomy in patients with ERM, MH, and rhegmatogenous retinal detachment (RRD).[14] Fujikawa et al. reported an increase in the IOP after vitrectomy in eyes with a MH during a long-term follow-up but not in eyes with an ERM.[15] These differences may be related to the type of vitreoretinal disease.

Vitrectomy for an ERM is thought to be the least traumatic type of vitrectomy. Thus, the effects of the vitrectomy in eyes with ERM could serve as a control for the effects of vitrectomy procedures on the rise of the IOP. On the other hand, vitrectomy for RRD is known to cause more inflammation from the dispersion of pigment granules from the retinal pigment epithelium (RPE) into the vitreous cavity preoperatively and also by the intraoperative procedures, e.g., laser photocoagulation and gas or silicone oil tamponade.

Eyes that have undergone vitrectomy for MH can also serve as controls for the effects of complete fluid-gas exchange and maintaining a face down position after surgery because both MH and RRD patients need complete fluid-gas/silicone oil exchange and are instructed to maintain face down position after surgery. These factors could influence the postoperative IOP.[16, 17]

The purpose of our study was to determine the long-term IOP changes following vitrectomy and to investigate the relationship between the type of retinal disease undergoing vitrectomy and the incidence of late-onset IOP elevation.

## Patients and methods

### Ethics statement

The procedures used in this study conformed to the tenets of the Declaration of Helsinki. This was a retrospective, observational, comparative, single-center study, and the procedures were approved by the Institutional Review Board and the Ethics Committee of the Nagoya University Graduate School of Medicine. An informed consent had been obtained from all of the patients for the surgery after an explanation of the procedures to be performed and possible complications. Permission was also obtained to use the data collected for future research.

### Subjects

We reviewed the medical records of all patients who had undergone 20-, 23- or 25-gauge PPV for RRD, ERM, or MH at the Nagoya University Hospital from January 2008 to June 2013. All patients had received a comprehensive ophthalmic examination including the measurements of the IOP with a non-contact tonometer (NT-530P, Nidek. Co. Ltd., Aichi, Japan), and measurements of the best-corrected visual acuity (BCVA) and the axial length. In addition, the anterior segment and the fundus were examined by slit-lamp biomicroscopy and ophthalmoscopy. All measurements and examinations were made before and 1, 3, 6, and 12 months following the surgery and at the final visit (average duration, 23.9 months). We also assessed the preoperative and postoperative lens status, macula status, the effect of combined cataract surgery, internal limiting membrane (ILM) peeling, the use of triamcinolone acetonide (TA) to make the vitreous and internal limiting membrane (ILM) more visible, and the type of retinal tamponade. A postoperative increase in the IOP by ≥4 mmHg relative to the baseline was defined as a significant IOP elevation. In addition, a rise in the IOP by ≥4 mmHg was defined as a ‘death’ event for the Kaplan-Meier survival analyses.

### Exclusion criteria

Patients with a preoperative diagnosis of glaucoma, ocular hypertension, asthma and chronic obstructive pulmonary disease which are treated with steroid or ipratropium bromide,[18] or diabetes regardless of the presence of diabetic retinopathy were excluded. Patients who had a history of vitrectomy, buckling surgery, ocular inflammation, penetrating ocular trauma, ocular ischemia, or long-term topical steroid medication were also excluded. Patients with RRD who had undergone simultaneous encircling, buckling surgery, silicone oil tamponade, or reoperation were also excluded. Patients followed for less than one year after the surgery were excluded.

### Surgical techniques

Standard 3-port PPV was performed with either 20-, 23- or 25-gauge instruments after retrobulbar anesthesia with 2.5 ml of 2% lidocaine and 2.5 ml of 0.5% bupivacaine.

Cataract surgery was performed in eyes with lens opacities or with a narrow angle. For the surgery, a 2.4-mm wide, self-sealing sclerocorneal tunnel was created at 12 o’clock, and a continuous curvilinear capsulorhexis was made. The nucleus of the lens was removed, and the residual cortex was aspirated with an irrigation/aspiration tip. Next, a foldable acrylic IOL was implanted in the lens bag.

A trocar was inserted at an angle of approximately 30 degrees to the limbus with the bevel-side up for the 23- and 25-gauge vitrectomy. After making the 3 ports, vitrectomy was performed using the Accurus or Constellation system (Alcon Inc., Fort Worth, TX). The required procedures for each disease was performed, e.g., complete peripheral vitreous removed, fluid-air exchange, and intraoperative photocoagulation for a RRD, The ERM was removed in eyes with an ERM, and the ILM was peeled with fluid-air exchange for eyes with a MH. After the fluid-air exchange, 20% sulfur hexafluoride (SF_6_) or 12% octafluoropropane (C_3_F_8_) gas was injected into the vitreous to tamponade the retina if needed. After the IOP was adjusted to a normal tension, the cannulas were withdrawn. At the end of surgery, gentamicin and betamethasone were injected subconjunctivally. Topical administration of betamethasone and anti-bacterial drops were administered four times daily for 3 months. Patients with RRD or MH were instructed to maintain a face down position for 1 week postoperatively.

### Statistical analyses

Statistical analyses were performed with the IBM SPSS Statistics for Windows, Version 22.0 (IBM Corp., Armonk, NY). Chi-square tests were used to compare the categorical data including preoperative macula status and the incidence of fibrin formation, and Kruskal-Wallis tests were used to compare continuous variables. Repeated analysis of variance with post-hoc Bonferroni corrections was used to evaluate the IOP changes. Kaplan-Meier survival analysis with log-rank test was used to compare the groups. Stepwise multivariate logistic regression analysis was used to assess the risk factors for postoperative IOP elevation using the preoperative IOP, BCVA, diagnosis, axial length, operative procedure, operation time, vitreous tamponade, and postoperative lens status.

## Results

The baseline demographics of all of the patients are shown in Table 1. Fifty-four eyes of 54 patients with RRD, 117 eyes of 117 patients with ERM, and 75 eyes of 75 patients with MH were studied. The mean age was 57.4 ± 10.9 years in the RRD group, 66.5 ± 10.4 years in the ERM group, and 63.4 ± 7.7 years in the MH group. The average follow-up time at the final visit was 2.0 ± 1.3 years with a range of 1.0 to 6.0 years in the RRD group, 1.9 ± 1.3 years with a range of 1.0 to 6.2 years in the ERM group, and 2.1 ± 1.3 years with a range of 1.0 to 6.6 years in the MH group. There were significant differences in the age (*P* <0.001), the sex distribution (*P* <0.001), the axial length (*P* <0.001), and the preoperative BCVA (*P* <0.001) among the groups.

**Table 1:** Patients demographic characteristics.

Characteristic	RRD (n = 54)	ERM (n = 117)	MH (n = 75)	*P*-value
Age (years)	57.4 ± 10.9	66.5 ± 10.4	63.4 ± 7.7	< 0.001
Men/Women	36/18	43/74	26/49	< 0.001
Axial length (mm)	25.6 ± 2.0	24.0 ± 1.7	24.3 ± 1.5	< 0.001
Follow-up period (years)	2.0 ± 1.3	1.9 ± 1.3	2.1±1.3	0.305
BCVA (logMAR)	0.96 ± 0.92	0.34 ± 0.23	0.65 ± 0.23	< 0.001
Preoperative macula status (on/off)	24/30	-	-	-

RRD = rhegmatogenous retinal detachment, ERM = epi-retinal membrane, MH = macular hole, BCVA = best corrected visual acuity, P-value determined by Kruskal-Wallis test in age, axial length, follow-up period, and BCVA.

P-value determined by Chi-Square test in gender.

The surgical procedures used for each patient are shown in Table 2. The preoperative lens status was not significantly different among the groups. There were significant differences in the ratio of eyes that had simultaneous cataract surgery (*P* = 0.009), ILM peeling (*P* <0.001), use of TA (*P* <0.001), and vitreous tamponade (*P* <0.001) among the 3 groups. The eyes in the ERM group and MH group were more likely to have undergone simultaneous cataract surgery, ILM peeling, and the use of TA. The ERM group had a tendency not to use long acting vitreous gas tamponade. However, there was no significant difference in the number of eyes that had a vitreous tamponade between the RRD group and the MH group.

**Table 2:** Patients surgical characteristics.

Characteristic		RRD	ERM	MH	*P*-value
Preoperative lens status					0.302
	Phakia	51	107	70	
	IOL	2	10	5	
	Aphakia	1	0	0	
Cataract surgery					0.009
	Combined PEA+IOL	37	97	67	
	Vitrectomy alone	17	20	8	
ILM peeling					< 0.001
	+	8	110	74	
	-	46	7	1	
TA usage					< 0.001
	+	40	101	73	
	-	14	16	1	
Vitreous tamponade					< 0.001
	None	0	71	0	
	Air	2	42	1	
	SF_6_	47	4	61	
	C_3_F_8_	5	0	13	
Gauge of vitrectomy					0.046
	20-gauge	25	46	45	
	23-gauge	14	39	20	
	25-gauge	15	32	10	

RRD = rhegmatogenous retinal detachment, ERM = epi-retinal membrane, MH = macular hole, BCVA = best corrected visual acuity.

P-value derived from Chi-square test.

**Fig 1:**
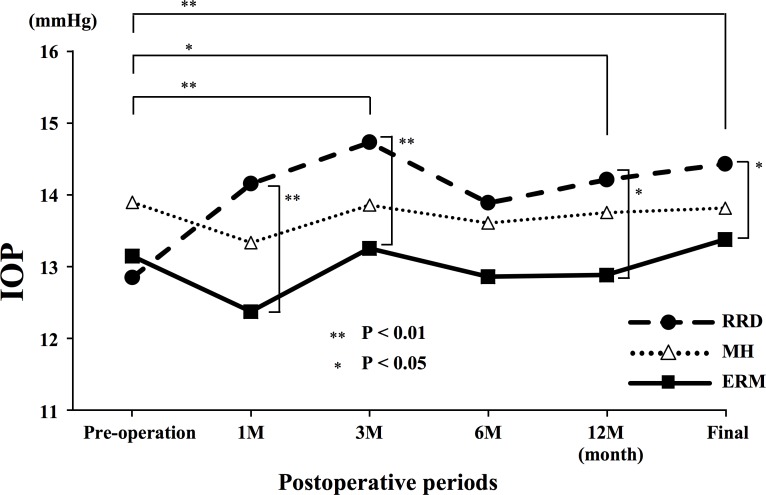
The mean intraocular pressures (IOP) at different postoperative times in eyes with rhegmatogenous retinal detachment (RRD), epi-retinal membrane (ERM), and macular hole (MH). The mean IOP at 3 and 12 months following surgery, and at the final visit were significantly higher than that before surgery in the RRD group. The IOP in the RRD group was significantly higher than that in the ERM group at 1, 3 and 12 months after surgery and at the final visit. ** *P* <0.01, *P <0.05

In the RRD group, the mean IOP was 12.9 ± 3.3 mmHg before surgery and 14.2 ± 3.0 at 1 month, 14.7 ± 3.1 at 3 months, 13.9 ± 2.7 at 6 months, 14.2 ± 2.5 mmHg at 12 months, and 14.4 ± 2.9 mmHg at the final visit. The mean IOP at 3 and 12 months and at the final visit were significantly higher than that before surgery (*P* = 0.001, *P* = 0.011, and *P* = 0.002, respectively; Fig 1). There were no significant differences in the incidence of postoperative IOP elevation between the macula-on and macula-off groups. The mean postoperative BCVA at the final visit was significantly improved from 0.96 ± 0.92 to 0.20 ± 0.44, and there was no significant difference in the final BCVA between eyes with an IOP increase of ≥4 mmHg from the preoperative IOP and without IOP elevation.

In the ERM group, the mean IOP was 13.1 ± 2.7 mmHg before surgery, 12.4 ± 3.1 at 1 month, 13.3 ± 3.0 at 3 months, 12.9 ± 2.8 at 6 months, 12.9 ± 2.6 mmHg at 12 months, and 13.4 ± 2.8 mmHg at the final visit. There was no significant change in the mean IOPs before and at any time following surgery.

In the MH group, the mean IOP was 13.9 ± 2.8 mmHg before surgery and 13.3 ± 3.4 at 1 month, 13.9 ± 2.9 at 3 months, 13.6 ± 3.1 at 6 months, 13.8 ± 2.8 mmHg at 12 months following surgery, and 13.8 ± 3.1 mmHg at the final visit. There were no significant changes in the IOPs at any time following surgery.

The mean IOP in the RRD group was significantly higher than that in the ERM group at 1 (*P* = 0.005), 3 (*P* = 0.009), and 12 months (*P* = 0.013) following surgery and at the final visit (*P* = 0.032; Fig 1). There were no significant differences in the IOP changes among the eyes that had 20–23- or 25-gauge surgery. Fibrin formation was observed in 5 eyes of RRD group within 3 days after the surgery, and it then disappeared spontaneously without posterior synechia. The incidence of formation was not statistically correlated with the IOP elevation.

**Fig 2:**
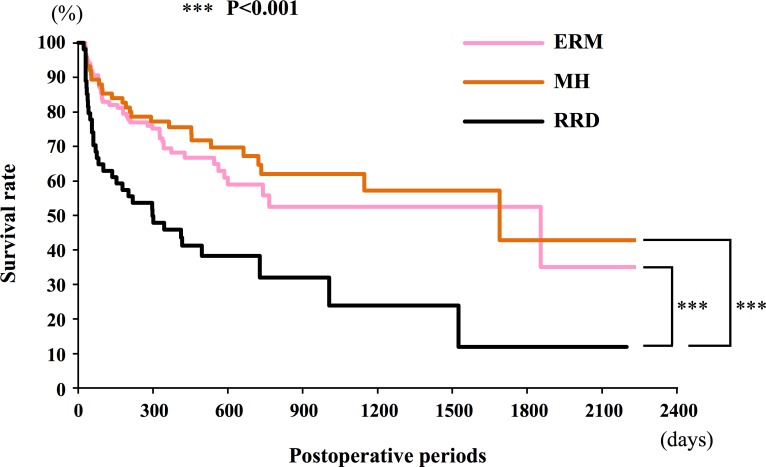
Kaplan-Meier survival curves for the rhegmatogenous retinal detachment (RRD), epi-retinal membrane (ERM), and macular hole (MH) groups. The cumulative proportion of eyes with a postoperative intraocular pressure increase by ≥4 mmHg from the baseline. The RRD group has significantly higher risk of IOP increase after vitrectomy compared to the ERM and the MH group (*P* <0.001 by log-rank test).

Kaplan-Meier survival curves indicated that the RRD group had a significantly higher risk of an IOP increase following vitrectomy than the ERM and the MH groups (*P* <0.001, log-rank test; Fig 2).

In each group, a low preoperative IOP was the risk factor that was significantly associated with a postoperative IOP elevation (RRD, odds ratio, 0.747, *P* = 0.02; ERM, odds ratio,0.794, *P* = 0.006; MH, odds ratio, 0.768, *P* = 0.014). A preoperative diagnosis of RRD was the only risk factor that was significantly associated with a postoperative IOP elevation after excluding eyes with a low preoperative IOP (odds ratio, 3.208; *P* = 0.003; Table 3).

**Table 3:** Multivariate logistic regression analysis.

Variable		Odds ratio (95% CI)	*P*-value
Preoperative IOP		0.780 (0.695–0.876)	<0.001
Diagnosis			0.006
	ERM	1.000	
	MH	0.933 (0.489–1.782)	0.834
	RRD	3.208 (1.480–6.953)	0.003
Axial length		Not included	0.177
BCVA		Not included	0.623
Concurrent cataract surgery		Not included	0.375
Operation time		Not included	0.552
Tamponade		Not included	0.361
Gauge		Not included	0.663
Postoperative lens status		Not included	0.311

CI = confidence interval, ERM = epi-retinal membrane, MH = macular hole

RRD = rhegmatogenous retinal detachment.

P-value derived from stepwise multivariate logistic regression analysis.

## Discussion

Our results showed that a late-onset IOP elevation was observed following vitrectomy only in the RRD group. The mean IOP in the RRD group was higher than that in the ERM group at 1, 3, and 12 months following surgery and at the final visit. In addition, Kaplan-Meier analyses showed that eyes in the RRD group had a higher risk of an IOP increase ≥4.0 mmHg than those in the other groups. Logistic regression analyses showed that a diagnosis of RRD triples the odds ratio of developing a rise in the IOP over eyes with an ERM.

We evaluated the postoperative IOP changes in eyes that undergone conventional surgery for RRD, ERM, and MH groups for at least one year. The IOP in the ERM group, which is thought to be the least invasive vitrectomy in this study, did not increase significantly at any time following vitrectomy. Fujikawa et al. also reported that the mean IOP in eyes with ERM that had undergone vitrectomy did not increase significantly during a mean follow-up period of 29.3 months.[15] These results indicate that vitrectomy can cause late-onset IOP elevation, but most of eyes that undergo vitrectomy do not have late-onset IOP elevation following vitrectomy.

On the other hand, a significant IOP elevation was observed following vitrectomy only in the RRD group. A preoperative diagnosis of RRD was the only significant risk factor for a postoperative IOP elevation even excluding eyes with a low preoperative IOP. It is known that eyes with an RRD tend to have lower IOP before surgery. However, our results suggest that the pathophysiology of RRD and the procedures to treat a RRD can cause late-onset IOP elevation following the vitrectomy.

The exact mechanism that contributes to the late-onset ocular hypertension following vitrectomy has not been determined. The mechanisms that contributes to the elevated IOP are most likely multiple and complex, and they are usually related to an increase in the resistance to outflow. The results of several recent studies have suggested that vitrectomy increases the oxygen tension within the eye; the partial oxygen pressure is highly elevated in the vitreous cavity especially posterior to the lens following vitrectomy because the vitreous lacks a diffusion barrier for the oxygen.[19–21] This elevated partial oxygen pressure may lead to increased oxygen stress on the trabecular meshwork causing the IOP elevation.[9] We removed the peripheral vitreous completely in eyes with a RRD, but did not for ERM and MH because core vitrectomy should be enough for these two macular diseases. However, it would result in a lack of an oxygen gradient in the vitreous and increasing oxygen stress to the trabecular meshwork in eyes with RRD which may cause the late-onset IOP elevation.

We have studied eyes that underwent trabeculectomy for glaucoma or uncontrollable ocular hypertension with topical medication following vitrectomy and found that 60% of the eyes had diabetes retinopathy (unpublished data). It has been reported that the presence of diabetes has a potential risk for IOP elevation [22] because of the oxidative stress on the trabecular meshwork.[9, 23] Thus, to avoid confounding factors, we excluded patients with diabetes.

It has been reported that lens extraction was a strong risk factor for the development of late-onset OAG after uncomplicated PPV. [9, 10] The RRD group had a larger number of eyes that had lens sparing vitrectomy than the other groups because the age of this group was relatively younger. Also, there was no significant correlation between the postoperative lens status and the IOP elevation in the RRD group (*P* = 0.566, data not shown). However, the RRD group had a significant lower rate of combined cataract and vitrectomy surgery. Accordingly, it is less likely that the combined surgery affected the IOP elevation in the RRD group.

A recent study suggested that a gas tamponade might cause an IOP increase in the long term. [15] A gas tamponade causes an increase in the concentrations of inflammatory cytokines or a clogging of the trabecular meshwork with inflammatory cells after fluid–gas exchange. The prone position would exacerbate this.[15] On the other hand, another study found that IOP was not significantly different between eyes receiving gas tamponade and eyes not receiving gas tamponade after vitrectomy combined with cataract surgery for up to 5 years. [24] In our study, there was no significant difference in the incidence of late-onset ocular hypertension between eyes with a MH in which gas tamponade was used, and eyes with an ERM in which gas tamponade was not used. These results suggest that it is less likely that gas tamponade caused the late-onset IOP elevation.

Laser photocoagulation was used to treat the retinal break(s) for all of the cases in the RRD group, and RPE cells were probably dispersed into the vitreous cavity. The IOP elevation could be caused by a clogging of the trabecular meshwork by these RPE cells.

We should also consider the effects of residual TA on the IOP elevation. TA can remain longer in the vitreal cavity following vitrectomy. In our study, TA was used in fewer eyes in the RRD group, and a late-onset IOP elevation was observed in the RRD group. These results suggest that the TA that was used during the vitrectomy was probably not responsible for the late-onset IOP elevation following the vitrectomy.

Our study has some limitations. First, this was a retrospective study, and the postoperative flare and the level of intraocular inflammatory cytokines had not been examined during the time postoperative times in these patients. Second, the sex and age were not matched among the groups. Third, we used topical administration of betamethasone as anti-inflammation for first 3 months after surgery. It cannot be denied that the use of betamethasone would cause an increase of IOP during this period. However, betamethasone was used on all of the eyes which should result in no difference among the groups in the incidence of IOP elevation especially in the long follow-up period. Prospective studies with investigation of the postoperative intraocular inflammation, longer term follow-up, sex, and age-match cohorts will be necessary to determine the exact mechanism(s) for the IOP elevation.

In conclusion, our results showed that a late-onset IOP elevation was observed only in eyes with RRD following vitrectomy. The elevation could be caused by the specific character of RRD and the surgical procedures. Thus, we should pay attention to the IOP elevation for long period following vitrectomy especially in eyes with RRD to prevent from the development of OAG.

## Supporting Information

S1 Dataset (XLSX)
